# Comparison Between the Effect of the Information–Motivation–Behavioral (IMB) Model and Psychoeducational Counseling on Sexual Satisfaction and Contraception Method Used Under the Coercion of the Spouse in Iranian Women: A Randomized, Clinical Trial

**DOI:** 10.1055/s-0043-1772487

**Published:** 2023-09-08

**Authors:** Alieh Zarbaf, Atefeh Ahmadi, Elahe Rafati, Faezeh Ghorbani, Masumeh Ghazanfar Pour, Katayoun Alidousti

**Affiliations:** 1 Kerman University of Medical Sciences, Kerman, Iran

**Keywords:** psychoeducational counseling, information–motivation–behavioral model, sexual satisfaction, contraception violence, aconselhamento psicoeducativo, modelo de informação-motivação-comportamental, satisfação sexual, violência contraceptiva

## Abstract

**Objective** Women play an essential role in maintaining the family's health, and family planning is part of women's and families' health. The couple's mutual understanding of family planning methods is essential in selecting contraception. Acceptance of and satisfaction with different contraception methods can impact sexual satisfaction. The present study aimed to compare the effect of the information-motivation-behavioral (IMB) model and psychoeducational counseling on sexual satisfaction and contraception methods of women referring to health centers in Kerman.

**Methods** This trial was conducted on 81 women aged 18 to 45, in Kerman health centers, from 2021 to 2022. Participants were randomly divided into 3 groups of 27 people (control, psychoeducational counseling, and IMB method). Three online counseling sessions were held for the psychoeducational group, and four were held for the IMB group. The control group received routine care. The IBM SPSS Statistics for Windows, version 22 (IBM Corp. Armonk, NY, USA) was used for data analysis using nonparametric Friedman and Kruskal-Wallis tests.

**Results** The mean age of participants was 32.59 ± 7.04, and the majority of them had university degrees and were homemakers. The mean sexual satisfaction score significantly increased immediately after the intervention and 1 month later in the 2 interventional groups ( *p* < 0.0). Changes in contraception methods after intervention were significant in the psychoeducational group ( *p* = 0.0)

**Conclusion** The results indicate the positive impact of psychological counseling on women's sexual satisfaction and contraception method. The IMB method also impacted men's sexual satisfaction but did not lead to any changes in the contraceptive method.

## Resumo

**Objetivo** A mulher desempenha um papel essencial na manutenção da saúde da família, e o planejamento familiar faz parte da saúde da mulher e da família. A compreensão mútua do casal sobre os métodos de planejamento familiar é essencial na seleção da contracepção. A aceitação e a satisfação com os diferentes métodos contraceptivos podem afetar a satisfação sexual. O presente estudo teve como objetivo comparar o efeito do modelo informação-motivação-comportamental (IMB) e aconselhamento psicoeducacional sobre a satisfação sexual e métodos contraceptivos de mulheres encaminhadas para centros de saúde em Kerman.

**Métodos** Este estudo foi realizado em 81 mulheres de 18 a 45 anos, nos centros de saúde de Kerman, de 2021 a 2022. As participantes foram divididas aleatoriamente em 3 grupos de 27 pessoas (controle, aconselhamento psicoeducacional e método IMB). Foram realizadas três sessões de aconselhamento online para o grupo psicoeducativo e quatro para o grupo IMB. O grupo de controle recebeu cuidados de rotina. O *IBM SPSS Statistics for Windows* , versão 22 (IBM Corp. Armonk, NY, EUA) foi utilizado para a análise dos dados por meio dos testes não paramétricos de Friedman e Kruskal-Wallis.

**Resultados** A média de idade das participantes foi de 32,59 ± 7,04, sendo que a maioria delas possuía nível superior e eram donas de casa. A pontuação média de satisfação sexual aumentou significativamente imediatamente após a intervenção e 1 mês depois nos 2 grupos de intervenção (p <0,0). As mudanças nos métodos contraceptivos após a intervenção foram significativas no grupo psicoeducativo (p = 0,0)

**Conclusão** Os resultados indicam o impacto positivo do acompanhamento psicológico na satisfação sexual das mulheres e no método contraceptivo. O método IMB também impactou a satisfação sexual dos homens, mas não levou a nenhuma mudança no método contraceptivo.

**Clinical trial registration:**https://fa.irct.ir/ Iranian Registry of Clinical Trial (IRCT20151103024866N16).

## Introduction

Women play an essential role in family maintenance, which, ultimately, has a a direct impact in the community's health; therefore, the community's health is affected when there is a threat to a woman's health. 1 Family planning is a part of comprehensive reproductive health and one of the most basic and essential healthcare system programs. 2 Contraception methods are essential in preventing unintended pregnancies, achieving the desired number of children and the proper spacing between pregnancies, and preventing high-risk pregnancies, unsafe abortions, maternal and neonatal mortality, and sexually-transmitted infections. 3 The diversity of contraceptive options for women, the limited methods available for men, and the existence of misconceptions, including gender attitudes, which consider family planning to be solely the responsibility of women, have led men to participate less in family planning programs. 4 The lack of couples' shared participation in using contraceptive methods is one of the areas of violence in reproductive health. Reproductive coercion is a behavior that interferes with the independent decision-making of a woman concerning reproductive health. It may take the form of pregnancy coercion, controlling the outcome of pregnancy, birth control sabotage, non-use of contraceptive methods, and forced use of a specific prevention method. The choice, acceptance, and satisfaction of women with different contraceptive methods affect their quality of life and sexual function, and different contraceptive methods have different effects on women's sexual satisfaction. 5 Sexual satisfaction is an essential indicator of sexual health and is strongly associated with empathy, love, emotions, creativity, and the frequency of sexual activity. Sexual satisfaction is obtained from positive sexual experiences. 6 Feelings of failure, frustration, and insecurity due to a lack of sexual satisfaction will likely endanger the mental health of spouses. 7 A review study by Maxwell et al. (2018) aimed at estimating the effect of intimate partner violence (IPV) on women's use of contraception showed that women who experienced IPV in the year prior to the study were 20% less likely to report the use of male condoms. 8

Intimate partner violence refers to behavior that causes physical, sexual, or mental pain, including acts of physical animosity, sexual constraint, mental mishandling, and controlling behaviors. 9 The non-participation of men in the use of contraceptive methods is one of the areas of violence in reproductive health. Violence has shown the highest correlation with six domains of reproductive health, including lack of use of contraceptive methods, abortion, reproductive system diseases, poor pregnancy outcomes, and lack of use of reproductive health services. 10

The empowerment of women with communication skills that allow them to face problems, choose the correct alternative behavior in problem-solving, and use family counseling services can be effective in preventing or reducing IPV. 11

One of the most comprehensive models for behavior change is the information-motivation-behavioral (IMB) skills model. 12 According to the model, health information, motivation, and behavioral skills are fundamental determinants of preventive behaviors and behavioral skills necessary for taking preventive measures. 13 The study by Mittal et al. (2017) aimed to present a supportive intervention to reduce HIV risk in women with a history of IPV. This supportive intervention included the key elements of the IMB model, the theory of gender and power (TGP) model, and family therapy. The results showed that safe sex and condom use increased at the end of the intervention. There was a significant reduction in violence and a significant improvement in self-esteem, anxiety, and posttraumatic stress disorder (PTSD). 13

Psychoeducational counseling is another type of counseling in which clients are trained during therapy. 14 Psychoeducational counseling for a particular situation or disease means providing the patient with the necessary information to create a new mental and cognitive understanding of what they have just encountered and helping them change their behavior; this is an essential component of every psychotherapy program. 15 A study by Akbarinejad et al. (2016) investigated the effect of psychoeducational group counseling on the postnatal sexual intimacy of lactating women. Results showed the positive impact of group counseling on the sexual intimacy of women after their first birth in the intervention group and increased sexual intimacy in this group. 14 Psychoeducational counseling is associated with education during therapy, and another feature of this type of counseling is its emphasis on prevention. Given that one of the structures of the IMB model is based on knowledge and cognition, both methods—psychoeducational counseling and IMB—have a psychological and social approach. In face of the mentioned issues, a study was conducted to compare the effect of the information–motivation–behavioral (IMB) model and psychoeducational counseling on sexual satisfaction and contraception method used under the coercion of the spouse in Iranian women.

## Methods

**Fig. 1:**
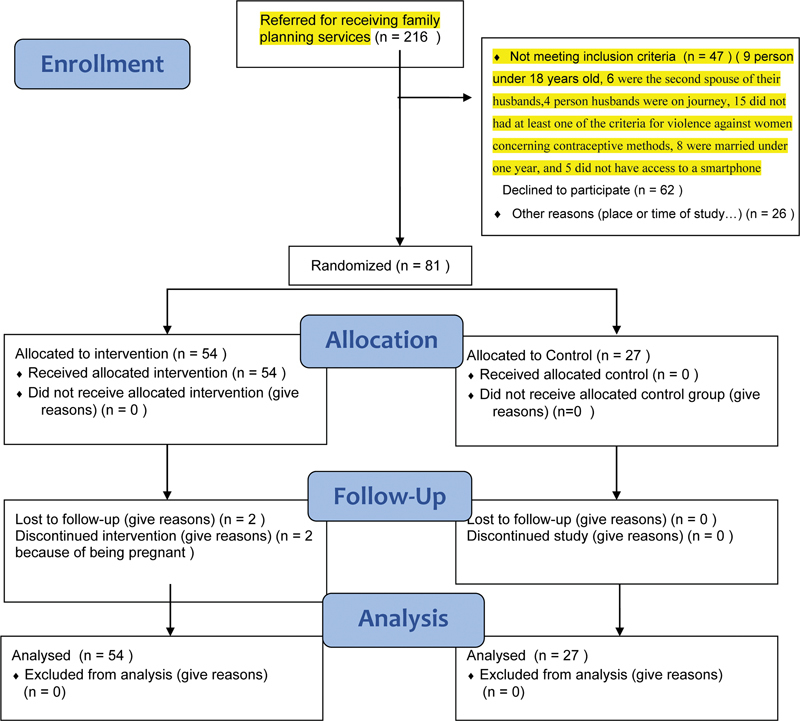
CONSORT 2010 flow diagram.

This study was a clinical trial (IRCT20151103024866N16), and the statistical population included all married women aged 18 to 45 years who were referred to health centers in Kerman, a city in the south of IRAN; to receive care and family planning counseling. The ethical committee of the university approved the study, and all women signed an informed consent before enrollment. Convenience sampling was used for participants who were women whose husbands did not cooperate in choosing a contraceptive method but complained and made excuses about every method the woman used. These women were under the coercion of spouses in contraception use, according to World Health Organization (WHO) guidelines (refusal of specific contraceptive methods, or insistence on a particular type of method, or resistance to contraceptive counseling, history of repeated pregnancies, or request for a medical termination, and insistence on tubal ligation or insistence on reversal of tubal ligation). 16 Based on the available sampling, each woman who applied for a contraceptive was asked the WHO guideline questions, and if she was under the pressure of her husband to receive a contraceptive and met the inclusion criteria, she was selected. The purpose of the research was explained to these women, and if they were satisfied and willing, they would enter the study ( Fig. 1 ).

The sample size needed to achieve a reliability of 1.96 and study power of 85%, based on the results of the study by Nabavi et al. (2019), was approximately 6 participants for each group; however, 27 people were selected to increase the study capacity and compensate for the loss of samples. 17

Because the samples were divided into 3 groups, the final sample size was 81 people. The identified women were included in the study if they consented to participate and had the inclusion criteria. Then, all identified persons were randomly divided through a table of random numbers into three groups: control, psychoeducational counseling, and the IMB model.

The inclusion criteria were married women in Kerman aged between 18 and 45, who were the only spouse of their husbands and whose spouses were present in Kerman during the intervention; had at least one of the criteria for violence against women regarding contraceptive methods according to the checklist of the WHO; consented to participate in the study; were literate; had been married for at least 1 year; had no known mental illness; and had access to a smartphone (due to online education) and the ability to use it. The exclusion criteria included pregnancy or participating in other psychological counseling classes simultaneously. Reasons for discontinuation were absence in two or more of the counseling sessions, and unwillingness to continue participation.

The research tool consisted of demographic information, a checklist for evaluating the contraception method requested by the spouse (WHO), and a special researcher-made questionnaire on contraceptive methods and sexual satisfaction. This questionnaire was prepared based on scientific articles. 18 19 20 21 The special sexual satisfaction questionnaire examined contraceptive methods and sexual satisfaction with 48 items. The participants expressed their satisfaction with each item on a five-point Likert scale. The questionnaire was sent to expert professors to assess its validity, and the content validity was also determined quantitatively and qualitatively to determine the content validity of the questionnaire. The content validity index (CVI) and content validity ratio (CVR) were 0.93 and 0.98, respectively, and face validity was confirmed using experts' opinions. The questionnaire was then presented to 30 people from the target group to determine its face validity; then, internal consistency was determined using Cronbach α (0.855).

First, the study's objectives were explained to women who met the inclusion criteria, and, if they wished to participate, written informed consent was obtained from them. All three groups completed the questionnaire on sexual satisfaction, specific contraception methods, and contraception type before the intervention, immediately after, and 1 month after the intervention. The study's objectives were first fully explained to participants to prevent information exchange between group members. Introduction sessions were held separately for each group. Given that information exchange is possible in cyberspace, to prevent information exchange between participants, after dividing them into 3 groups (control, psychoeducational, and IMB), a time interval of 2 months was considered for each group. First, a pretest was completed for the control group, and posttests were done 1 month later and 1 month after the initial posttest. Then a pretest was done for the psychoeducational group; the initial posttest was done after the three virtual counseling sessions in Skyroom; the final posttest was completed 1 month after the intervention. The total time spent implementing the intervention and completing the questionnaires was 6 months. In the control group, the clinic midwife provided all routine training. In the intervention group, psychoeducational counseling sessions were held according to a unique package of counseling sessions in 3 90-minute online sessions 1 week apart. In the IMB model intervention group, counseling sessions were held according to a specific package, in 4 online sessions, 120 to 190 minutes each 2 sessions per week, and all these sessions were held by the same person (the researcher). Finally, immediately after the intervention and 1 month later, the participants in the 3 groups completed the questionnaire. The psychoeducational counseling and IMB program packages were designed, prepared, and implemented using various resources 216 people were surveyed to participate in the study, of which 135 were either not eligible or unwilling to participate in the study. Eighty-one people were included in the study. By lot, they were divided into three groups (one was a control group and two were intervention groups). Since the intervention was performed on and offline, we did not have any sample drop, and, finally, the analysis was performed on 81 people. Data were analyzed using the IBM SPSS Statistics for Windows, version 22 software (IBM Corp. Armonk, NY, USA). Quantitative variables were described by mean and standard deviation, and qualitative variables were defined by frequency and frequency percentage. The Kolmogorov-Smirnov and Shapiro-Wilk tests were used to evaluate the normality of dependent variables (sexual satisfaction and changes in contraceptive methods). Due to the abnormality of data distribution, the Friedman test was used to examine the trend of changes. The nonparametric equivalent of one-way analysis of variance, Kruskal-Wallis, was used for comparison ( Chart 1 ).

**Chart 1:** Psychoeducational counseling content and information–motivation–behavioral model

Session	Objective	Content
Psychoeducational counseling package		
First	Introduction and awareness	Informing people about violent behaviors, teaching contraception methods
Second	Identifying sexual misconceptions	Teaching to improve sexual relations, expressing the importance of sex, talking about sexual misconceptions, extensive training on contraception methods
Third	Effective communication and assertiveness	Assertiveness skill training, communication styles, effective communication skills training
Information–motivation–behavioral package		
First	Identifying distorted dimensions in sex	Defining contraception methods and the benefits and harms of each method, defining spousal violence in contraception, defining sexual satisfaction and sexual satisfaction related to contraception.
Second	Motivational dialogue	Conducting a motivational interview to accept or change the contraception method to make it voluntary and increase sexual satisfaction
Third	Efficient sexual dialogue with the spouse	Improving perceived individual skills and self-efficacy
Fourth	Activate assertive behaviors	Activating avoidance behaviors and improving assertiveness skills by increasing motivation and behavioral skills

## Results

The mean age of participants was 32.59 ± 7.04. There was no statistically significant difference between the three groups regarding age, education, occupation, breastfeeding, weight, number of pregnancies, etc. The three groups were similar in demographic characteristics ( Table 1 ).

**Table 1:** Comparison of the distribution of qualitative and quantitative demographic variables between the intervention and control groups

Variables		Group			*P* -value *
		Psychoeducation N (%)	IMB N (%)	Control N (%)	
Women education	High school education	2 (7.4%)	2 (7.4%)	3 (10%)	0.9
	Diploma	13 (48.1%)	13 (48.1%)	11 (36.7%)	
	University education	12 (44.4%)	12 (44.4%)	16 (53.3%)	
Women job	Housekeeper	19 (70.4%)	15 (55.6%)	20 (66.7%)	0.34
	Employee	6 (22.2%)	7 (25.9%)	9 (30%)	
	self-employment	2 (7.4%)	5 (18.5%)	1 (3.3%)	
Spouse education	High school education	3 (11.1%)	7 (25.9%)	5 (18.5%)	0.22
	Diploma	12 (44.4%)	13 (48.1%)	9 (30%)	
	University education	12 (44.4%)	7 (25.9%)	16 (53.3%)	
Spouse job	permanent job	15 (55.6%)	13 (48.1%)	14 (46.7%)	0.83
	Temporary job	10 (37%)	13 (48.1%)	15 (55.6%)	
	workless	2 (7.4%)	1 (3.3%)	1 (3.3%)	
**Variable**		**Mean ± SD**	**Mean ± SD**	**Mean ± SD**	***P*** -value **
Age		7.255 ± 34.44	7.704 ± 32.04	31.30 ± 6.199	0.22
Age of onset of sexual activity		4.029 ± 23.19	3.652 ± 21.52	3.151 ± 22.27	0.24
Parity		1.368 ± 2.89	1.812 ± 2.85	1.654 ± 2.77	0.89

Abbreviation: IMB, information-motivation-behavioral.

* Chi-square.

** Kruskal-Wallis test.

The results showed that the mean sexual satisfaction score immediately after the intervention was statistically significant between the three groups ( *p* < 0.01). According to the Kruskal-Wallis test, the mean sexual satisfaction score 1 month after the intervention was significantly different among the three groups ( *p* < 0.01). The sexual satisfaction score increased 1 month after the intervention in the psychoeducational and IMB group, and the increase in sexual satisfaction was more significant in the IMB group ( Table 2 ).

**Table 2:** Mean and standard deviation of sexual satisfaction score before the intervention, after the intervention and one month after the intervention three groups

Sexual satisfaction	Mean ± SD			*P* -value *
	Psychoeducation	IMB	Control	
Before intervention	22.73 ± 166.66	18.43 ± 167.62	18.08 ± 169.33	0.97
After intervention	16.85 ± 184.37	13.01 ± 190.29	15.38 ± 167.30	0.001<
One month after intervention	16.48 ± 189.25	11.05 ± 203.48	15.49 ± 166.13	0.001<
*P* -value **	0.001<	0.001<	0.71	

Abbreviation: IMB, information-motivation-behavioral.

* Kruskal-Wallis test.

** Friedman test.

The mean score of sexual satisfaction in the psychoeducational intervention group increased 1 month after the intervention compared to immediately after the intervention, and the difference was significant ( *p* = 0.03); in the IMB group, the sexual satisfaction increased significantly 1 month after the intervention compared to immediately after the intervention ( *p* < 0.01), but there was no statistically significant difference between the 2 intervention groups in sexual satisfaction ( *p* = 0.1). However, the mean score in the IMB group was higher than in the psychoeducational intervention group. Using each contraception method in the three groups (control, psychoeducational, and IMB intervention) was measured before, immediately after, and 1 month after the intervention. The Mann-Kendall statistical test showed that changes in the contraception method in the psychoeducational group were significant ( *p* = 0.02) ( Table 3 ).

**Table 3:** Frequency distribution of contraceptive methods before intervention, after intervention and one month after intervention in three groups

Group	Contraceptive method	Before intervention	After intervention	One month after intervention	*P* -value *
Psychoeducation	Withdrawal	14 (51.85%)	8 (29.62%)	8 (29.62%)	0.02
	Condom	6 (22.22%)	11 (40.74%)	11 (40.74%)	
	Combined oral pills	5 (18.51%)	4 (14.81%)	4 (14.81%)	
	Medroxyprogesterone asetat	1 (3.7%)	2 (7.4%)	2 (7.4%)	
	IUD	1 (3.7%)	2 (7.4%)	2 (7.4%)	
IMB	Withdrawal	13 (48.14%)	10 (37.03%)	5 (18.51%)	0.07
	Condom	5 (18.51%)	8 (29.62%)	9 (33.33%)	
	Combined oral pills	5 (18.51%)	5 (18.51%)	6 (22.22%)	
	Medroxyprogesterone asetat	1 (3.7%)	1 (3.7%)	2 (7.4%)	
	IUD	3 (11.11%)	3 (11.11%)	5 (18.51%)	
Control	Withdrawal	14 (51.85%)	14 (51.85%)	15 (50%)	1
	Condom	9 (33.33%)	9 (33.33%)	8 (29.62%)	
	Combined oral pills	5 (18.51%)	5 (18.51%)	5 (18.51%)	
	Medroxyprogesterone asetat	1 (3.7%)	1 (3.7%)	2 (7.4%)	
	IUD	1 (3.7%)	1 (3.7%)	1 (3.7%)	

Abbreviation: IMB, information-motivation-behavioral; IUD, intrauterine device.

* Kendall test.

## Discussion

According to the present study results, the sexual satisfaction level in the two intervention groups increased significantly, which shows that both psychoeducational counseling and IMB counseling increased women's sexual satisfaction.

In the study by Alirezaei et al. (2022), the sexual satisfaction of infertile couples increased after psychological intervention, which was consistent with our study. 22 However, due to the long duration of psychological intervention (6 months) compared to IMB counseling (2 weeks) and psychoeducational counseling (3 weeks), it seems that the counseling methods in the present study provide a more appropriate interpretation. Our study is consistent with that of Akbar Nejd et al. (2020), which showed the positive impact of psychoeducational group counseling on the sexual intimacy of lactating women, leading to an increase in sexual intimacy. 14 Considering that sexual intimacy is itself a component in increased sexual satisfaction and improvement in the quality of marital life, it can be concluded that psychoeducational training can raise sexual satisfaction and improve other effective details of sexual satisfaction. The study results by Tahan et al. (2020) showed that women's sexual satisfaction increased after receiving psychoeducational counseling. 23 In the study by Bober et al. (2015), the sexual psychological intervention increased sexual desire, female sexual satisfaction, and female sexual self-efficacy by increasing the sexual information of women with ovarian cancer. 24 In the study by Ali Mohammadi et al. (2018), counseling based on sexual self-efficacy on sexual functioning and sexual satisfaction of newly married women showed that sexual self-efficacy counseling had an effect on sexual functioning but did not affect sexual satisfaction, which was not consistent with our results. 25 It can be concluded that IMB counseling has a higher impact on sexual satisfaction than sexual self-efficacy counseling, despite fewer sessions.

In the present study, the mean score of sexual satisfaction in the two psychoeducational and IMB interventions increased after counseling. Psychoeducational counseling and training on sexual issues and contraception methods can improve marital quality, such as sexual satisfaction, sexual intimacy, and marital satisfaction, and increase the use of safe contraception methods. The IMB approach is also a pattern of behavior change and consists of three components. It helps couples obtain the necessary information about sexual issues and contraception methods. They will be able to acquire appropriate behavioral skills in dealing with the spouse and choosing the proper contraception method. A comprehensive counseling approach can identify women's sexual needs, which leads to improved behavior and change in women's behavior to promote sexual satisfaction.

The results of the study by Cavallaro et al. (2020) show that women who received systematic counseling on family planning methods continued to use contraception methods, and interruption of contraceptive use was lower than in the control group, which was consistent with the results of psychoeducational counseling in our study. 26 In a study by Jiang et al. (2019), which examined the predictors of condom use in Chinese gay men based on the modified IMB model, the results showed that using the modified IMB model directly contributes to safe sexual behaviors and leads to increased use of condoms. The results of this study were not consistent with the IMB model in our study. 27 In a survey by Fullerton et al. (2013) on the effect of the IMB model concerning condom use and hormonal methods of contraception as well as the use of both ways simultaneously (dual protection), the results showed that the components of the IMB model support the sexual health of young women and also contribute to dual protective behaviors and the prevention of sexually transmitted infections and pregnancies, which was not consistent with our study. 28 According to the studies, the IMB model leads to the use of safe contraception methods and the prevention of high-risk behaviors. However, in our research, the ineffectiveness of IMB counseling in significantly changing couples' contraception method choice could be due to simultaneous training about sexual satisfaction and contraception methods. The couples were in stable and permanent relationships, and the purpose of this study is to increase sexual satisfaction related to contraception methods.

Choosing and accepting and being satisfied with different contraceptive methods can affect the quality of life and sexual performance of women. Choosing a contraceptive method by husband coercion can cause the non-continuation of using the method or incorrect use, which will result in unwanted pregnancy and illegal abortions and complications. 29

According to the results, it can be concluded that women whose spouses coerce them to use specific contraception methods not only need to change their contraception methods but increasing their knowledge about contraceptive methods sometimes leads to their complete acceptance. So, it can create positive relationships between partners, they were able to come to an agreement with their spouse in selecting the method of contraception and realized that the new contraceptive method chosen by both was the most appropriate method of contraception for them. After agreeing on the new contraceptive method and improving interpersonal relationships with their spouse, their sexual satisfaction also increased. Because the study was conducted during the coronavirus disease 2019 (COVID-19) outbreak and the sessions were given online, women who were under the coercion of spouses were able to participate in the training course without leaving home and benefit from the counseling courses without facing any resistance from their husbands, which can be considered as the strength of the study. Due to the need for access to smartphones to attend online courses, people from certain social and economic classes could not participate in the counseling courses, which can be considered a limitation of the present study.

## Conclusion

Psychological counseling could improve women's sexual satisfaction and lead to change in the contraception method, in cases on which it was not according to the women's wishes. The results also showed that the IMB method positively impacted women's sexual satisfaction but had no impact on changing the contraceptive method. Using appropriate contraception to prevent unwanted pregnancy is one of the essential parts of reproductive health, and utilization of intervention methods seems crucial. According to the results, using one of these two intervention methods in contraceptive counseling sessions is good.

## References

[1] Khani S Hamzeh Gardeshi Z Bozorgi N A Review on Various Aspects of Male Involvement in Women's Sexual and Reproductive Health J Mazandaran Univ Med Sci 2017 27 152 99 116 http://jmums.mazums.ac.ir/article-1-9430-en.html

[2] Abedi G Takbiri F Akberi S Rostami F Phenomenology of Perception and Behavior of Women in Safe Methods of Family Planning: A Qualitative Study Majallah-i Zanan, Mamai va Nazai-i Iran 2012 15 33 1 9 10.22038/ijogi.2012.260

[3] Duru C B Andibanbang F A Duru C A Ogelle O M Contraceptive Prevalence, Pattern and Socio-demographic Determinants among In- Union Women of Reproductive Age (15–49 years) in Semi-urban Communities of Orlu town, Imo state, Nigeria. JDDT [Internet]15 Aug. 2022 [cited 30Apr.2023];12(4-S):68–2. Available from:https://jddtonline.info/index.php/jddt/article/view/5511

[4] KHalajabadi F F Heidari J Male participation in family planning in Zanjan, 2011: a qualitative study Hakim Research Journal 2013 16 2383 3742 https://www.sid.ir/en/journal/ViewPaper.aspx?ID=339356

[5] Grace K T Anderson J C Reproductive Coercion: A Systematic Review Trauma Violence Abuse 2018 19 04 371 390 10.1177/1524838016663935 27535921 PMC5577387

[6] Shindel A W Baazeem A Eardley I Coleman E Sexual Health in Undergraduate Medical Education: Existing and Future Needs and Platforms J Sex Med 2016 13 07 1013 1026 10.1016/j.jsxm.2016.04.069 27318019

[7] Setoudeh S Motaghi M Mousavi M Survey of sexual satisfaction in women referred to public health centers of Mashhad Journal of Sabzevar University of Medical Sciences. 2017 26 01 73 80

[8] Maxwell L Brahmbhatt H Ndyanabo A Wagman J Nakigozi G Kaufman J S The impact of intimate partner violence on women's contraceptive use: Evidence from the Rakai Community Cohort Study in Rakai, Uganda Soc Sci Med 2018 209 25 32 10.1016/j.socscimed.2018.04.050 29783092

[9] https://apps.who.int/violence-info/intimate-partner-violence/

[10] Nia A SN Dolatian M Hasan Pour Azghadi B Ebadi A Akbarzadeh Baghban A Domestic violence and its association with domains of reproductive health in women: A systematic review J Mazandaran Univ Med Sci 2018 27 158 205 217

[11] Sarichloo M E GS KZ MBM Jahani-Hashemi H Effectiveness of health education interventions to prevent domestic violence against women J Soc Welfare 11 237 257

[12] Osborn C Y Rivet Amico K Fisher W A Egede L E Fisher J D An information-motivation-behavioral skills analysis of diet and exercise behavior in Puerto Ricans with diabetes J Health Psychol 2010 15 08 1201 1213 10.1177/1359105310364173 20453056 PMC3086815

[13] Mittal M Thevenet-Morrison K Landau J Cai X Gibson L Schroeder A An integrated HIV risk reduction intervention for women with a history of intimate partner violence: Pilot test results AIDS Behav 2017 21 08 2219 2232 10.1007/s10461-016-1427-5 27172976 PMC6390962

[14] Akbarinejad Z Alidoosti K Ghorashi Z Asadollahi Z The effect of psychoeducational group counseling on postnatal sexual intimacy of lactating women referring to urban health centers in Rafsanjan City: An educational trial JRUMS 2020 18 10 969 984 http://journal.rums.ac.ir/article-1-4715-en.html

[15] Saisto T Toivanen R Salmela-Aro K Halmesmäki E Aoeg S Therapeutic group psychoeducation and relaxation in treating fear of childbirth Acta Obstet Gynecol Scand 2006 85 11 1315 1319 10.1080/00016340600756920 17091410

[16] World Health Organization 2014 Health care for women subjected to intimate partner violence or sexual violence: a clinical handbook World Health Organization https://apps.who.int/iris/handle/10665/136101

[17] Nabavi S J Sanai Zaker B Kiyamanesh A Evaluation of efficacy of relationship enhancement and McMaster approach on adjustment of couples Medical Sciences 2018 28 04 319 324 http://tmuj.iautmu.ac.ir/article-1-1493-en.html

[18] Rabieepur S Ebrahimi M Sadeghi E Relationship between Sexual Health and Contraception Methods in Women J Mazandaran Univ Med Sci 2015 25 130 30 39 http://jmums.mazums.ac.ir/article-1-6390-en.html

[19] Higgins J A Smith N K The Sexual Acceptability of Contraception: Reviewing the Literature and Building a New Concept J Sex Res 2016 53(4-5):417 456 10.1080/00224499.2015.1134425 26954608 PMC4868075

[20] Bahrami N Soleimani M A Shraifnia H Masoodi R Shaigan H Zh M rezaei Female Sexual Satisfaction with Different Contraceptive Methods IJN 2012 25 76 55 63 http://ijn.iums.ac.ir/article-1-1284-en.html

[21] Banai M Beheshti M Shah Rahmani H Sexual and Reproductive Health Pregnancy and Postpartum. First, editor: Artin Teb; 1397. 196 p. Pardell-Dominguez L., Palmieri P.A. Dominguez-Cancino, K.A. et al. The meaning of postpartum sexual health for women living in Spain: a phenomenological inquiry BMC Pregnancy Childbirth 2021 21 92 10.1186/s12884-021-03578-y PMC7844957 33509133

[22] Alirezaei S Taghipour A Latifnejad Roudsari R The effect of infertility counseling interventions on marital and sexual satisfaction of infertile couples: A systematic review and meta-analysis Int J Reprod Biomed (Yazd) 2022 20 10 795 806. Published 2022 Nov 2 10.18502/ijrm.v20i10.12264 PMC9644646 36381353

[23] Tahan M Saleem T Moshtagh M Fattahi P Rahimi R Psychoeducational Group Therapy for sexual function and marital satisfaction in Iranian couples with sexual dysfunction disorder Heliyon 2020 6 07 e04586 10.1016/j.heliyon.2020.e04586 32775734 PMC7394864

[24] Bober S L Recklitis C J Bakan J Garber J E Patenaude A F Tjosm J Addressing sexual dysfunction after risk-reducing salpingo-oophorectomy: effects of a brief, psychosexual intervention J Sex Med 2015 12 01 189 197 10.1111/jsm.12713 25311333 PMC4304978

[25] Alimohammadi L Mirghafourvand M Zarei F Pirzeh R The effectiveness of group counseling based on Bandura's self-efficacy theory on sexual function and sexual satisfaction in Iranian newlywed women: A randomized controlled trial Appl Nurs Res 2018 42 62 69 10.1016/j.apnr.2018.06.011 30029716

[26] Cavallaro F L Benova L Owolabi O O Ali M A systematic review of the effectiveness of counselling strategies for modern contraceptive methods: what works and what doesn't? BMJ Sex Reprod Health 2020 46 04 254 269 10.1136/bmjsrh-2019-200377 PMC7569400 31826883

[27] Jiang H Chen X Li J Tan Z Cheng W Yang Y Predictors of condom use behavior among men who have sex with men in China using a modified information-motivation-behavioral skills (IMB) model BMC Public Health 2019 19 01 261 10.1186/s12889-019-6593-8 30832640 PMC6399930

[28] Fullerton T Rye B Meaney G J Loomis C Condom and hormonal contraceptive use by young women: An information-motivation-behavioral skills assessment Canadian Journal of Behavioural Science 2013 45 03 196 10.1037/a0033309

[29] Lantiere A E Rojas M A Bisson C Fitch E Woodward A Stevenson E L Men's Involvement in Sexual and Reproductive Health Care and Decision Making in the Philippines: A Systematic Review of the Literature Am J Men Health 2022 16 04:15579883221106052 10.1177/15579883221106052 PMC9277450 35815925

